# Intravenous infusion of dexmedetomidine during the surgery to prevent postoperative delirium and postoperative cognitive dysfunction undergoing non-cardiac surgery: a meta-analysis of randomized controlled trials

**DOI:** 10.1186/s40001-024-01838-z

**Published:** 2024-04-18

**Authors:** Di Wang, Zhi Liu, Wenhui Zhang, Guo Zu, He Tao, Congjie Bi

**Affiliations:** 1 Department of Anaesthesiology, Dalian Municipal Central Hospital Affiliated to Dalian University of Technology, Dalian, Liaoning China; 2 China Medical University, Shenyang, China; 3 Dalian Medical University, Dalian, China; 4 Department of Gastroenterology, Dalian Municipal Central Hospital Affiliated to Dalian University of Technology, Dalian, Liaoning China

**Keywords:** Dexmedetomidine, Non-cardiac surgery and non-neurosurgery, Postoperative delirium, Postoperative cognitive dysfunction

## Abstract

**Background:**

Dexmedetomidine plays a pivotal role in mitigating postoperative delirium and cognitive dysfunction while enhancing the overall quality of life among surgical patients. Nevertheless, the influence of dexmedetomidine on such complications in various anaesthesia techniques remains inadequately explored. As such, in the present study, a meta-analysis was conducted to comprehensively evaluate its effects on postoperative delirium and cognitive dysfunction.

**Methods:**

A number of databases were searched for randomised controlled trials comparing intravenous dexmedetomidine to other interventions in preventing postoperative delirium and cognitive dysfunction in non-cardiac and non-neurosurgical patients. These databases included PubMed, Embase, and Cochrane Library. Statistical analysis and graphing were performed using Review Manager, STATA, the second version of the Cochrane risk-of-bias tool for randomised controlled trials, and GRADE profiler.

**Main results:**

This meta-analysis comprised a total of 24 randomised controlled trials, including 20 trials assessing postoperative delirium and 6 trials assessing postoperative cognitive dysfunction. Across these 24 studies, a statistically significant positive association was observed between intravenous administration of dexmedetomidine and a reduced incidence of postoperative delirium (RR: 0.55; 95% CI 0.47 to 0.64, *p* < 0.00001, *I*^2^ = 2%) and postoperative cognitive dysfunction (RR: 0.60; 95% CI 0.38 to 0.96, *p* = 0.03, *I*^2^ = 60%). Subgroup analysis did not reveal a significant difference in the incidence of postoperative delirium between the general anaesthesia and non-general anaesthesia groups, but a significant difference was observed in the incidence of postoperative cognitive dysfunction. Nonetheless, when the data were pooled, it was evident that the utilisation of dexmedetomidine was associated with an increased incidence of hypotension (RR: 1.42; 95% CI 1.08 to 1.86, *p* = 0.01, *I*^2^ = 0%) and bradycardia (RR: 1.66; 95% CI 1.23 to 2.26, *p* = 0.001, *I*^2^ = 0%) compared with other interventions. However, there was no significantly higher occurrence of hypertension in the DEX groups (RR = 1.35, 95% CI 0.81–2.24, *p* = 0.25, *I*^2^ = 0%).

**Conclusion:**

Compared with other interventions, intravenous dexmedetomidine infusion during non-cardiac and non-neurosurgical procedures may significantly reduce the risk of postoperative delirium and cognitive dysfunction. The results of subgroup analysis reveal a consistent preventive effect on postoperative delirium in both general and non-general anaesthesia groups. Meanwhile, continuous infusion during general anaesthesia was more effective in reducing the risk of cognitive dysfunction. Despite such findings, hypotension and bradycardia were more frequent in patients who received dexmedetomidine during surgery.

## Background

Postoperative delirium (POD) and postoperative cognitive dysfunction (POCD) are postoperative phenomena linked to a range of surgical complications. While some individuals argue that both POD and POCD can be collectively referred to as ‘postoperative cerebral dysfunction’, others distinguish POCD as a distinct condition from POD. Delirium, recognised as a common and severe acute neuropsychiatric syndrome, is primarily characterised by cognitive deficits and inattention. It also presents with alterations in arousal, disruptions in sleep–wake patterns, and other variations in mental state [1]. In a meta-analysis, the incidence of POD ranged from 8.4–18.1% in patients with total hip replacement and slightly higher 12.8–30.5% after total knee arthroplasty, depending on the patient population and the assessment means [2]^.^ In a study conducted in a tertiary care centre in Taipei, China, the prevalence of delirium was 5.1% among 701 patients (aged > 65 years) undergoing elective orthopaedic or urologic surgery [3]. Likewise, elderly patients who have undergone non-cardiac surgery frequently encounter POCD, which is frequently accompanied by a decline in cognitive function and an elevated risk of mortality. POD and POCD are significantly associated with adverse clinical outcomes, including higher mortality rates and increased complications. The research findings indicated that POD patients experienced a significantly higher mortality rate within one-year post-surgery compared to patients undergoing similar procedures. Specifically, the mortality rate among POD patients ranges from 17.4% to 22.7%, whereas the mortality rate for patients undergoing similar surgeries was only in the range of 7.5% to 8.4% [4, 5]. These conditions can inflict distress upon patients and their families, potentially leading to psychological challenges. Further, they can prolong hospital stays and result in escalated medical expenses. The association between POD and increased mortality may involve various mechanisms, including inflammatory response and immune system dysregulation, cognitive impairment, physical and psychological stress, neuroinflammatory changes, and the impact of sedatives and anaesthesia. These mechanisms are likely intertwined, collectively contributing to the occurrence of POD and heightened mortality risk. Monk’s study reported that among patients aged 60 and above who underwent non-cardiac surgery, the incidence of POCD was 41.4% on leaving the hospital and 12.7% at 3 months [6]. Several independent risk factors for early POCD were identified, including increased age, lower levels of education, longer duration of anaesthesia, postoperative infections, a history of previous surgery, and respiratory complications. Notably, only age was found to be a significant factor in long-term cognitive impairment following surgery [7]. POD is typically assessed using the Confusion Assessment Method (CAM). In contrast to POD, there is currently no universally accepted and standardised method for evaluating POCD within the medical community [8]. The Mini-Mental State Examination (MMSE) serves as a valuable tool for quantitatively assessing the extent of cognitive impairment and monitoring alterations in cognitive function. MMSE scores exhibit a positive correlation with the patient's cognitive capabilities [9].

Dexmedetomidine (DEX) is an alpha2-adrenergic agonist with high selectivity. It possesses the ability to suppress sympathetic excitability, enhance vagal excitability, lower blood pressure, reduce heart rate, and diminish myocardial oxygen consumption. Additionally, DEX exhibits sedative, analgesic, anxiolytic, hypnotic, amnesic, and narcotic effects. It reduces pain and anxiety by inhibiting neuronal firing, thereby achieving sedation. The pharmacological properties of dexmedetomidine include swift absorption, rapid distribution, quick metabolism, and prompt excretion. In clinical practice, dexmedetomidine can be used for sedation, analgesia and tranquillisation, as well as intra- and post-operative sedation and analgesia. Moreover, dexmedetomidine also inhibits the activity of the central nervous system, thereby reducing the patient’s pain. Several studies have reported a neuroprotective effect of DEX [10–12]. Despite previous studies having examined the impact of DEX on POD or POCD, owing to the lack of exclusion of confounding factors such as the type of surgery and anaesthesia protocol, the results have not always been applicable to clinical practice. As such, in the present study, a meta-analysis was conducted to evaluate the effectiveness of DEX in reducing the incidence of POD and POCD in patients undergoing non-cardiac surgery and non-neurosurgery. Further, a subgroup analysis was conducted based on the type of anaesthesia administered to account for any potential variations in results. As a secondary outcome, efforts were made to determine if dexmedetomidine affected the stability of the circulatory system.

## Methods

### Search strategy

A systematic search was conducted in the databases of PubMed, Embase, and The Cochrane Library to identify all relevant studies, spanning from their inception to February 10, 2023. Supplementary data were acquired from sources such as reference lists of the included articles or grey literature; however, no suitable literature was found for inclusion. The PubMed basic search strategy was as follows: (("delirium"[MeSH Terms] OR "delirium"[All Fields] OR ("Postoperative Cognitive Complications"[MeSH Terms] OR "Postoperative Cognitive Complications"[All Fields])) AND ("dexmedetomidine"[MeSH Terms] OR "dexmedetomidine"[All Fields])). The search strategy only included randomised controlled trials (RCTs), and the language was limited to English and Chinese. Species restriction was limited to human subjects. Di Wang, Wenhui Zhang, and Zhi Liu each performed independent literature searches, data extraction, and conducted bias and GRADE evaluation. In the event of any disagreements, the matter would be referred to the corresponding author for discussion and resolution. The present meta-analysis was prospectively registered in the PROSPERO database with the registration number CRD42023402004.

### Study selection

The inclusion criteria and requirements for the studies incorporated into this meta-analysis were as follows: (1) they were designed as randomised controlled trials (RCTs); (2) they were published in English or Chinese; (3) they involved patients undergoing non-cardiac surgery and non-neurosurgery procedures; and (4) the intervention included intravenous infusion of dexmedetomidine or the drugs used in the control group during the surgical procedure. The following were excluded from consideration: reviews, study protocols, observational studies, studies where the outcomes or adverse effects did not pertain to POD and POCD, studies involving critically ill patients or sedated patients in the ICU(ICU patients need complex care; multiple diseases, diverse medications, and interventions may complicate research reliability by confounding intervention effectiveness), studies where DEX was administered to patients through other routes, and duplicate or redundant reports.

### Endpoints

The primary outcome was the occurrence of POD and POCD during the seventh post-operative day, and the secondary outcomes were the occurrence of hypertension, hypotension, and bradycardia during the surgery.

### Data extraction

Data were extracted according to the standard forms, and any uncertainties or discrepancies were resolved through discussions with the corresponding author. The extracted data included the following: first author, publication year and country, experiment design, the sample size of the intervention and comparison groups, age range, and ASA Grade, anaesthetic type, surgery procedure, drugs applied in the control group, the anaesthesia depth monitoring, the strategy of DEX infusion, cognitive problem and the assessment method.

### Assessment of trial quality

The risk of bias was assessed using the risk bias assessment tool RoB2 (revised version 2019) for RCTs in six areas: the process of randomisation, departures from the intended interventions, lack of outcome data, measurement of the outcome, selection of the reported outcome, and overall risk of bias [13].

### Statistical analysis

For statistical analysis and graphing, Review Manager software (RevMan version 5.4.1), STATA software (Version 12.0), and GRADE profiler were used. Dichotomous data were analysed by using risk ratios (RR) with 95% confidence interval (CI). There were three levels of heterogeneity determined by the *I*^2^ statistics: low (*I*^2^ < 50%), moderate (50% ≤ *I*^2^ ≤ 75%), and high (*I*^2^ > 75%) [14]. When *I*^2^ < 50%, the fixed effect model was selected to pool the data; conversely, if a study was found to have moderate or high heterogeneity, the random effect model was selected to pool the data, and subgroup analysis was conducted to identify sources of heterogeneity. Publication bias was assessed by examining funnel plots and conducting Egger's test, with p-values greater than 0.05 indicating no significant publication bias. Additionally, a sensitivity analysis was performed to evaluate the stability of the results. Finally, the Grading of Recommendations Assessment, Development, and Evaluation (GRADE) technique was utilised to classify the major results of the degree of certainty.

## Results

### Search results

**Fig. 1:**
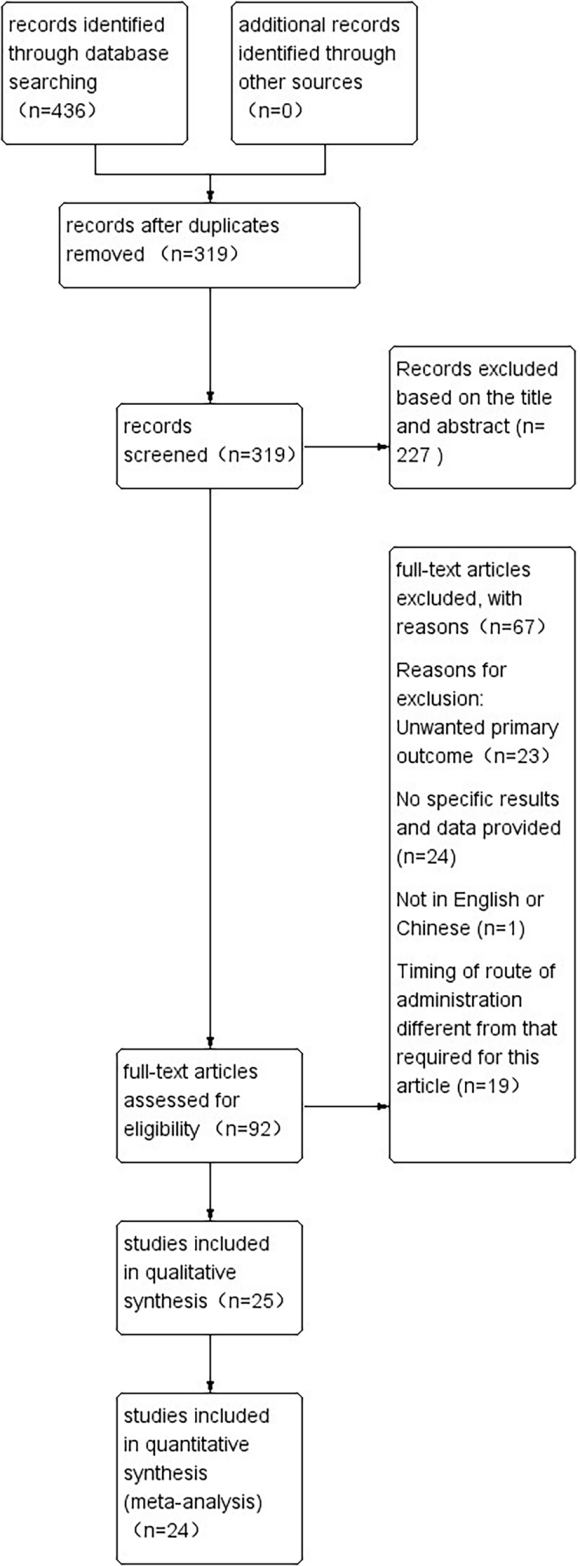
Flow diagram showing results of search and reasons for exclusion of studies

The process for searching and including literature in the database is illustrated in Fig. 1. After searching using the previously defined search strategy, a total of 436 articles were identified through the database search, while no additional articles were found through other sources. A total of 319 articles were retrieved after duplicated records were taken out. According to titles and abstracts, 227 of them were later excluded, mainly because of the low correlation between these studies and the aim of the meta-analysis. A full read-through of the other 92 articles was performed, and a further 67 articles were eliminated, as shown in Fig. 1. Ultimately, a total of 24 RCTs fully satisfied the inclusion criteria and were subsequently included in the meta-analysis (Fig. 1).

### Characteristics of trials

The present meta-analysis included trials published from 2014 to 2023, involving 5207 participants (2638 patients received intraoperative intravenous DEX for sedation and 2569 patients received other medication for sedation or saline as a control group). Notably, two studies [15, 16] evaluated both POD and POCD, and one study [17] employed two distinct methods to assess POCD, yielding different conclusions. Thus, these studies were included as two separate outcomes in the analysis. A total of 23 studies were reported in English and one study was published in Chinese. Among the included studies, there were 17 experiments conducted in China, 3 in Korea, 1 in Egypt, 1 in Japan, and 2 in India. In the present meta-analysis, a comprehensive examination involving 20 studies, with a total of 4304 patients, was conducted to investigate whether there exists an association between the use of DEX and a decrease in the incidence of POD. In addition, 7 studies, comprising 690 participants, were included to explore the potential impact of DEX on the incidence of POCD. In Table 1, detailed descriptions of the included trials are provided. All patients included in the study were over the age of 18 and drugs used in the control group included saline, midazolam and propofol. Table 1 also shows that the DEX doses and administration times were different. Although POCD evaluation instruments varied, the process for evaluating POD was generally consistent.

**Table 1:** The characteristics of the included studies

Study	Country	Design	Group	Anaesthetic type	Surgical procedure	Grade of ASA	Age range (years)	Control	Anaesthesia depth monitoring	Strategy of DEX	Cognitive problem	Assessment
Wu et al. [18]	China	2-arm RCT	DEX48 Control 48	General anaesthesia	Endoscopic sinus surgery	ASA I–II	20–30	NS	BIS	Load dose 0.5 μg kg − 1 over 10 min, followed by maintenance dose 0.2 ug /kg/ h	POD	NI
Shi et al. [16]	China	2-arm RCT	53/53	General anaesthesia	Thoracoscopic lobectomy with OLV	II -III	≥ 65	NS	BIS	0.5 µg/kg/h DEX intravenously from anaesthesia induction until chest closure	POD/POCD	CAM/comprehensive test scale
Tang et al. [15]	China	3-arm RCT	80/40	General anaesthesia	Hepatic lobectomy	II ~ III	60 ~ 80	NS	BIS	After anaesthesia induction, dexmedetomidine [0.3 μg/(kg·h)] in the Dex1 group, and dexmedetomidine [0.6 μg/(kg·h)] in the Dex2 group were infused until the end of operation	POD/POCD	CAM/MMSE
Cheng et al. [20]	China	2-arm RCT	23/22	General anaesthesia + modified intercostal nerve block	Single-port thoracoscopic surgery	II -III	18–75	NS	NI	An initial loading dose (0.4 μg/kg) was given for 15 min before the induction of anaesthesia, followed by a maintenance infusion of 0.4 μg/kg/h that was stopped 30 min before the end of surgery	POD	NI
Mishina et al. [30]	Japan	2-arm RCT	99/97	Local anaesthesia	Inguinal hernia repair	NI	20–85	midazolam	OAA/S	NI	POD	NI
Hu et al. [22]	China	2-arm RCT	77/75	General anaesthesia	Open transthoracic oesophagectomy	I–III	60–80	NS	BIS	A loading dose of dexmedetomidine, 0.4 ml /kg, bolus was administered over 15 min immediately prior to induction of anaesthesia, followed by a maintenance dexmedetomidine infusion of 0.1 ml/ kg/h until 1 h before the anticipated end of surgery	POD	CAM
Liu et al. [26]	China	4-arm RCT	99/98	General anaesthesia	Total hip joint or knee joint or shoulder joint replacement surgery	II–III	65–80	NS	BIS	A continuous infusion of DEX at 0.2–0.4 µg/kg/h, without an initial dose, was administered throughout the duration of the surgery	POD	CAM
Lu et al. [27]	China	2-arm RCT	344/331	General anaesthesia	Elective abdominal surgery	ASA I–III	60 +	NS	BIS	A loading dose of 0.5 μg/kg over 15 min followed by a maintenance dose of 0.2 μg/kg per hour)	POD	CAM
Liu et al. [25]	China	2-arm RCT	60/60	General anaesthesia	Oral and maxillofacial surgery	I–II	65 +	NS	BIS	At a dosage of 0.5 μg/kg for 10 min before anaesthesia induction, followed by continuous infusion at a rate of 0.4 μg/kg/h until half an hour before the end of surgery	POD	CAM
Wu et al. [33]	China	2-arm RCT	30/30	General anaesthesia	Thoracoscopic surgery	I–III	NI	NS	BIS	Receiving 0.5 μg/kg/h DEX infusion throughout surgery	POD	NI
Zhang et al. [35]	China	2-arm RCT	DEX120 Control 120	Subarachnoid block/Combined spinal–epidural anaesthesia/General anaesthesia	Hip fracture	I–III	65–90	NS	NR	(0.5 mg/kg/h) 30 min before the start of anaesthesia,and 0.3 mg/kg/h during the operation	POD	CAM
Xin et al. [34]	China	2-arm RCT	DEX30 Control 30	General anaesthesia	Laparoscopic cholecystectomy	II or III	65 +	NS	BIS	As a loading dose, 0.5 μg/kg dexmedetomidine was administered over 10 min before anaesthesia induction, which was then administered as a continuous intravenous infusion at a dose of 0.4 μg/kg/h until 30 min before the end of surgery	POD	3D-CAM
Mei et al. [28]	China	2-arm RCT	DEX148 Control 148	Lumbosacral plexus plus T12 paravertebral block	Hip arthroplasty	I–IV	65 +	propofol	BIS	Loading dose of DEX0.8–1 μg/kg in 15–20 min and followed by a continuous infusion (0.1–0.5 μg/ kg/h)	POD	CAM
Kim et al. [19]	Korean	2-arm RCT	60/60	General anaesthesia	Thoracoscopic lung resection surgery	I–III	18–75	NS	BIS	A fixed rate of 0.5 μg/kg/h until the end of the surgery	POD	CAM
Tang et al. [32]	China	2-arm RCT	54/52	General anaesthesia	Interventional embolisation	I to IV	18–70	NS	BIS	A loading dose of DEX (1.0 μg/kg) was administered for 15 min and then changed to a continuous infusion at 0.3 μg/kg/hand the infusion of DEX was stopped approximately 10 min before the end of surgery	POD	CAM-S
Chen et al. [20]	China	2-arm RCT	80/80	General anaesthesia	Elective cranial surgery	I–III	20 +	NS	BIS	An infusion of dexmedetomidine (0.5 μg /kg/ h) was started immediately before the induction of anaesthesia and was maintained throughout the surgery	POD	ICDSC
Shin et al. [31]	Korean	2-arm RCT	366/366	Spinal anaesthesia	Lower limb surgery	I–II	65 +	propofol	OAA/S	1 μg/kg dexmedetomidine was administered for more than 10 min as the loading dose, followed by continuous administration at 0.1 to 0.5 µg kg/ h	POD	CAM
Li et al. [24]	China	2-arm RCT	309/310	General anaesthesia	Major non-cardiac surgery	I–III	60 +	NS	BIS	A loading dose of dexmedetomidine 0⋅6 μg/kg 10 min before induction of anaesthesia followed by a continuous infusion (0⋅5 μg/ kg/ h) until 1 h before the end of surgery	POD	CAM
Mei et al. [29]	China	2-arm RCT	DEX183 Control 183	Spinal anaesthesia	Knee arthroplasty	I–IV	65 +	propofol	BIS	Loading dose of DEX0.8–1 μg/kg in 15–20 min and followed by a continuous infusion (0.1–0.5 μg/ kg/h)	POD	CAM
Lee et al. [23]	Korean	3-arm RCT	95/109	General anaesthesia	Laparoscopic major non-cardiac surgery	I to III	60 +	NS	BIS	received a dexmedetomidine 1 μg/kg bolus followed by 0.2–0.7 μg/kg/h infusion from induction of anaesthesia to the end of surgery	POD	CAM
Li et al. [37]	China	3-arm RCT	DEX55 Control 109	Combined spinal-epidural anaesthesia	Hip or knee replacement	I–III	65 +	Propofol/midazolam	BIS	NR	POCD	The neuropsychological test battery
Chawdhary et al. [36]	India	2-arm RCT	40/40	General anaesthesia	non-cardiac surgery	I–III	55 +	PROPOFOL	BIS	dexmedetomidine 0.5–0.7 µg/kg/h	POCD	neuropsychological tests
Mohamed et al. 1. [17]	Egypt	2-arm RCT	25/25	General anaesthesia	Abdominal surgery	I to III	60 +	NS	GE Entropy	at a dose of 0.4 μg/kg/h	POCD	Stroop colour word interference test
Mohamed et al. 2. [17]	Egypt	2-arm RCT	25/25	General anaesthesia	Abdominal surgery	I to III	60 +	NS	GE Entropy	at a dose of 0.4 μg/kg/h	POCD	MoCA
Nag et al. [38]	India	2-arm RCT	60/60	Spinal anaesthesia	Hip surgery	I–III	60–75	NS	NI	Dexmedetomidine injection at a dose of 1 mcg/kg over 10 min, after Spinal Anaesthesia and before start of surgery, followed by a continuous infusion at a rate of 0.4 mcg/kg/h until the end of surgery	POCD	MoCA

### Risk of bias in included studies

**Fig. 2:**
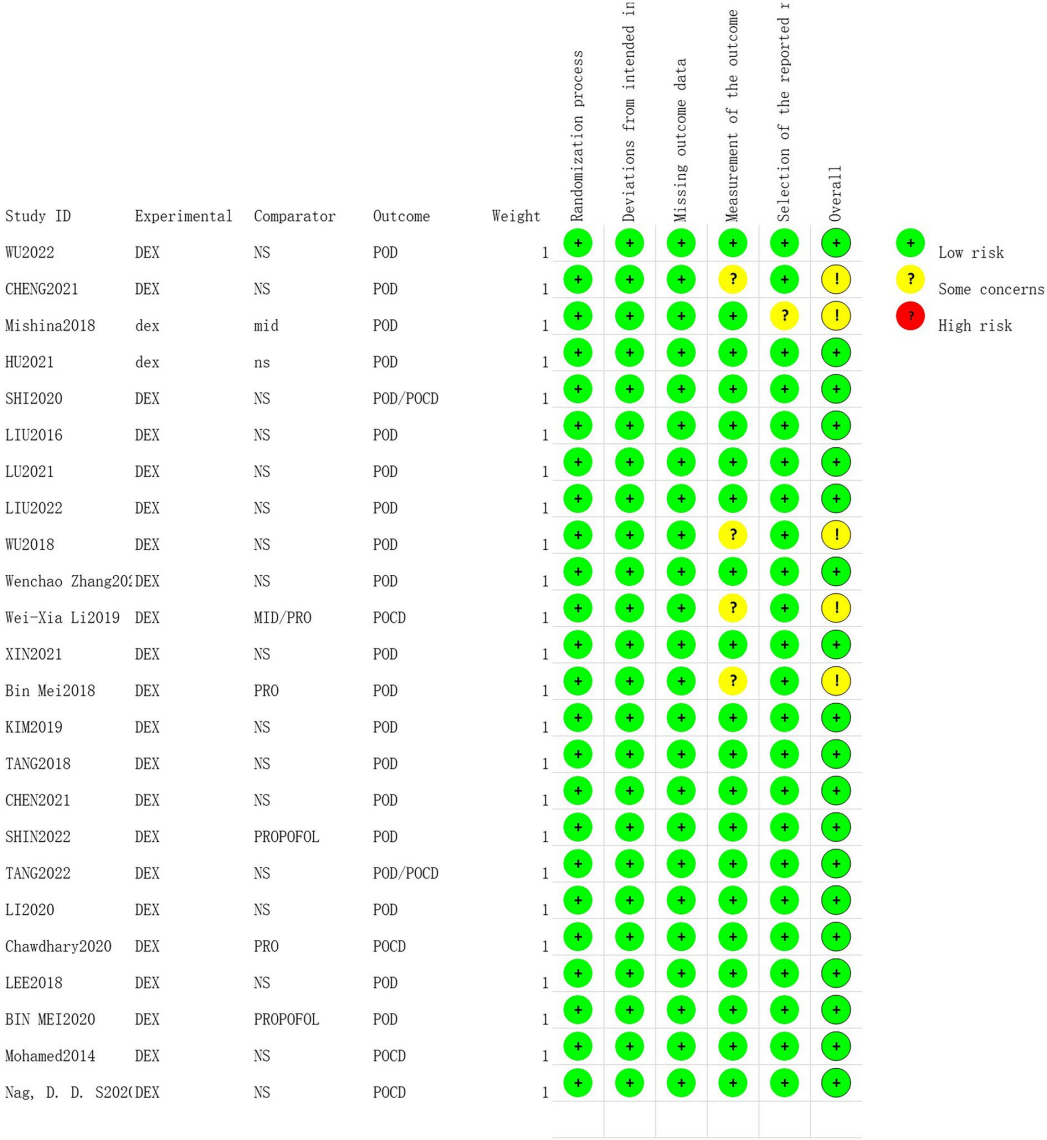
Quality assessment of each study included in the meta-analysis

Based on the main results, RoB 2.0 was used to assess 24 RCTs. The assessment revealed that 5 trials were determined to have ‘some concerns’, 19 trials were categorised as having a ‘low risk of bias’, and none of the trials were deemed to have a 'high risk of bias' (Fig. 2).

### Meta‐analysis for the studies on cognitive impairment

#### The occurrence of POD

**Fig. 3:**
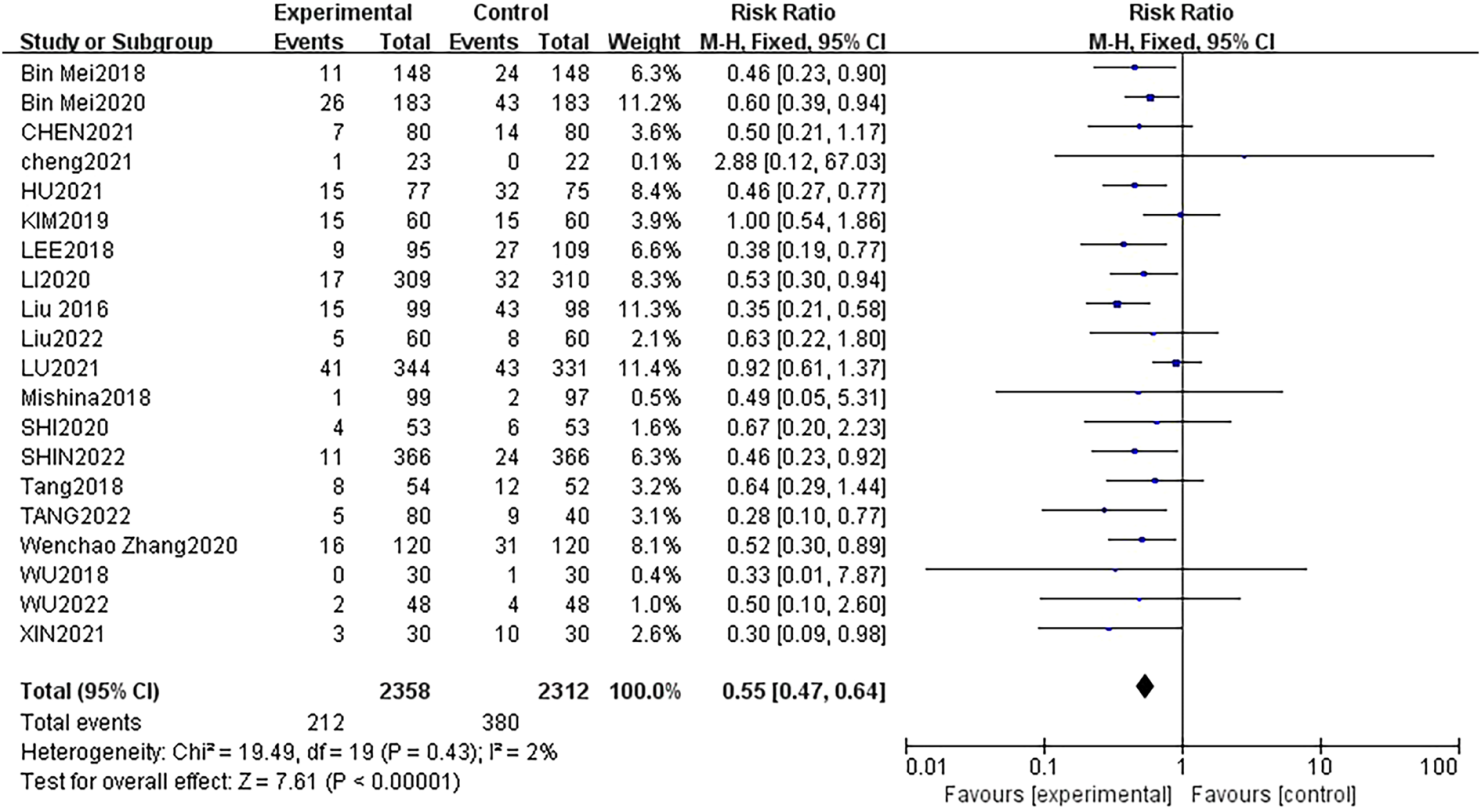
Forest plot of comparison: DEX vs control, outcome: incidence of POD

Twenty studies [15, 16, 18–35], with a total of 4304 patients, investigated the incidence of POD. There was no statistical heterogeneity between these studies (*P* = 0.43, *I*^2^ = 2%). A fixed-effects model was used for analysis, and the results indicated a statistically significant association. Specifically, in the context of non-cardiac surgery and non-neurosurgery procedures, the use of DEX was significantly linked to a reduced incidence of POD compared to the control group (19 trials, RR: 0.54; 95% CI 0.46 to 0.64; *p* < 0.0001) (Fig. 3).

#### The occurrence of POCD

**Fig. 4:**
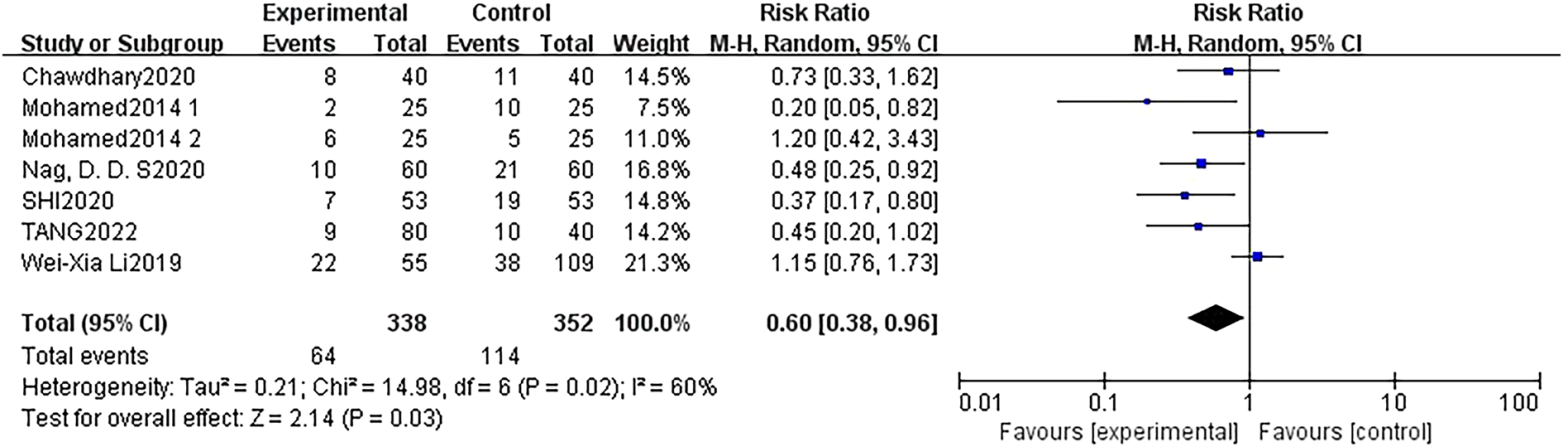
Forest plot of comparison: DEX vs control, outcome: incidence of POCD at 7 days after surgery

Six studies [15–17, 36–38] reported the occurrence of POCD on the seventh postoperative day. Various neuropsychological tests, including the Stroop colour word interference test, Montreal Cognitive Assessment (MoCA), and Mini-Mental State Examination (MMSE), were utilised as evaluation methods in these studies. One study [17] adopted two different methods for assessing POCD and reached different conclusions, and thus, were included in the analysis as two separate outcomes. The results of meta-analyses using a random-effects model revealed statistical differences in preventing POCD undergoing non-cardiac surgery and non-neurosurgery compared with the control group (7 trials, RR: 0.60; 95% CI 0.38 to 0.96, *p* = 0.03) with moderate heterogeneity (*P* = 0.02, *I*^2^ = 60%) (Fig. 4).

#### Secondary outcomes

**Fig. 5:**
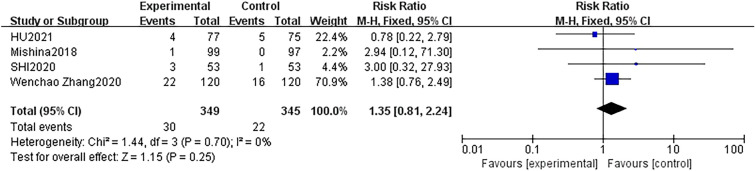
Forest plot of comparison: DEX vs control, outcome: incidence of hypertension

**Fig. 6:**
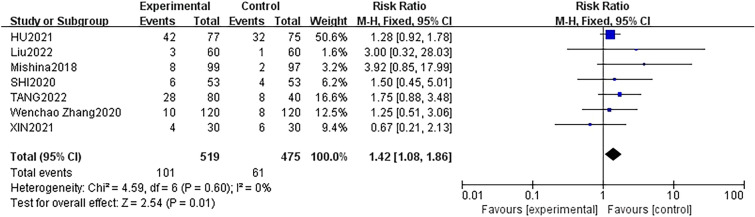
Forest plot of comparison: DEX vs control, outcome: incidence of hypotension

**Fig. 7:**
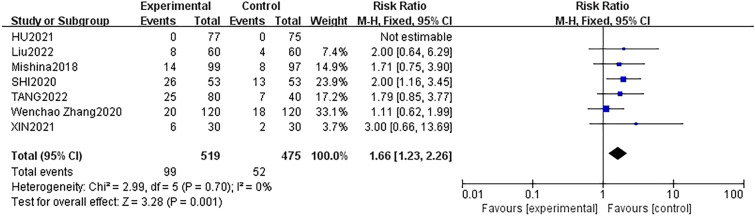
Forest plot of comparison: DEX vs control, outcome: incidence of bradycardia

Four studies [22, 25, 30, 35] with 694 participants (DEX group: 349 patients; control group: 345 patients) reported the occurrence of hypertension during surgery. The fixed effect model was applied, and no statistical heterogeneity was identified between studies (*P* = 0.70, *I*^2^ = 0%) in the present analysis. There was no significantly higher occurrence of hypertension in the DEX groups in the included studies (RR = 1.35, 95% CI 0.81–2.24, *p* = 0.25) (Fig. 5). Moreover, seven studies [15, 16, 22, 25, 30, 34, 35] with 994 patients (DEX group: 519 patients; control group: 475 patients) examined the occurrence of hypotension. These studies demonstrated that intravenous infusion of DEX for sedation led to intraoperative hypotension in patients when compared to the group that received other sedatives or normal saline, with low heterogeneity (RR: 1.42; 95% CI 1.08 to 1.86, *p* = 0.01, *I*^2^ = 0%) (Fig. 6). Likewise, these seven studies [15, 16, 22, 25, 30, 34, 35] also examined the occurrence of bradycardia. They revealed that intravenous infusion of DEX for sedation led to intraoperative bradycardia in patients when compared to the group that received other sedatives or normal saline, with low heterogeneity (RR: 1.66; 95% CI 1.23 to 2.26, *p* = 0.001, *I*^2^ = 0%) (Fig. 7).

### Subgroup analysis

**Fig. 8:**
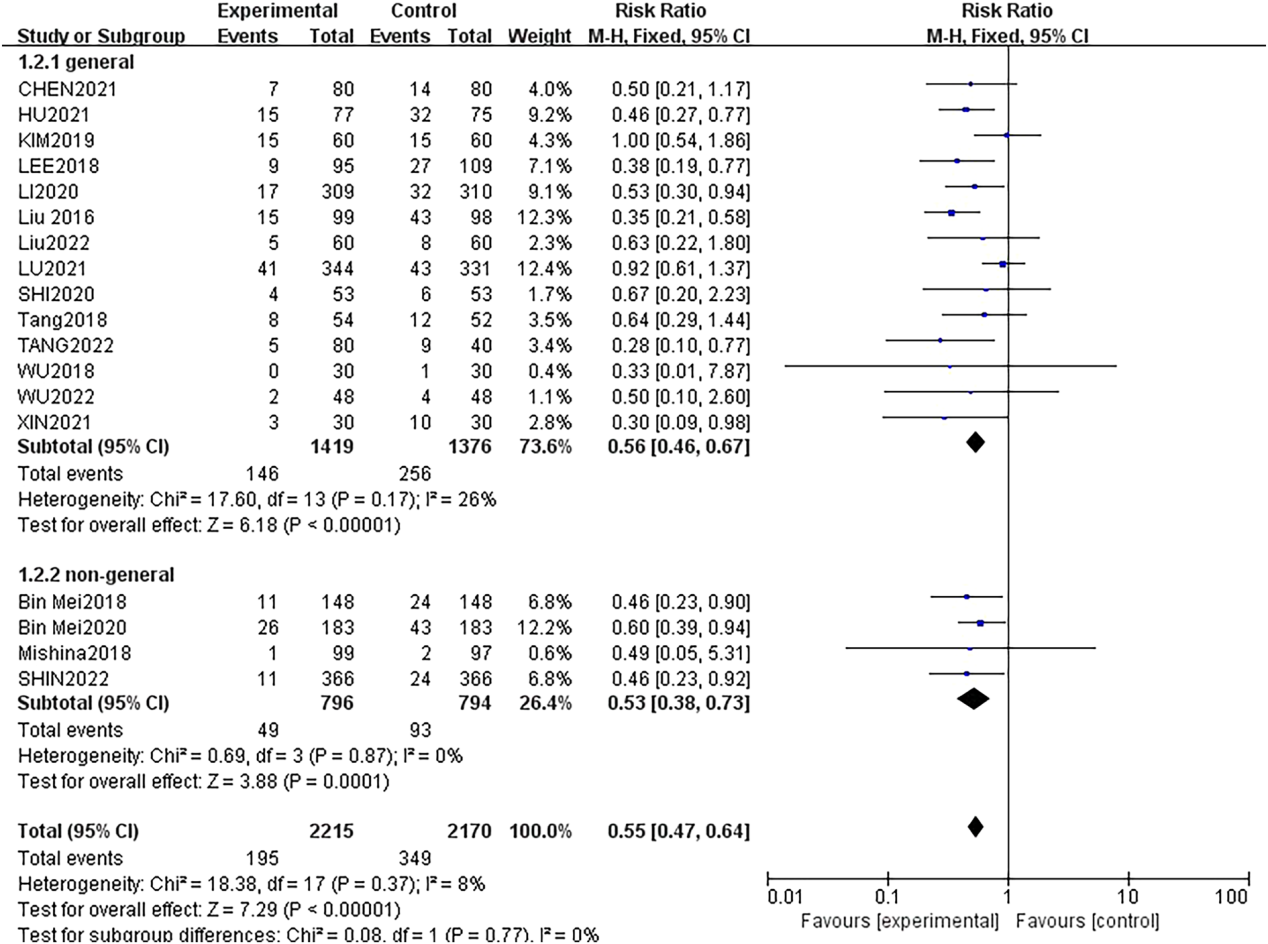
Forest plot of POD by subgroup based on the type of anaesthesia

The studies on the incidence of POD were categorised into two subgroups according to the type of anaesthesia. Fourteen studies were conducted with patients only receiving general anaesthesia, and four studies were tested under non-general anaesthesia plus sedation and assessed the effect on POD. Notably, regardless of the type of anaesthesia, DEX significantly reduced the prevalence of POD with low heterogeneity (Fig. 8).

**Fig. 9:**
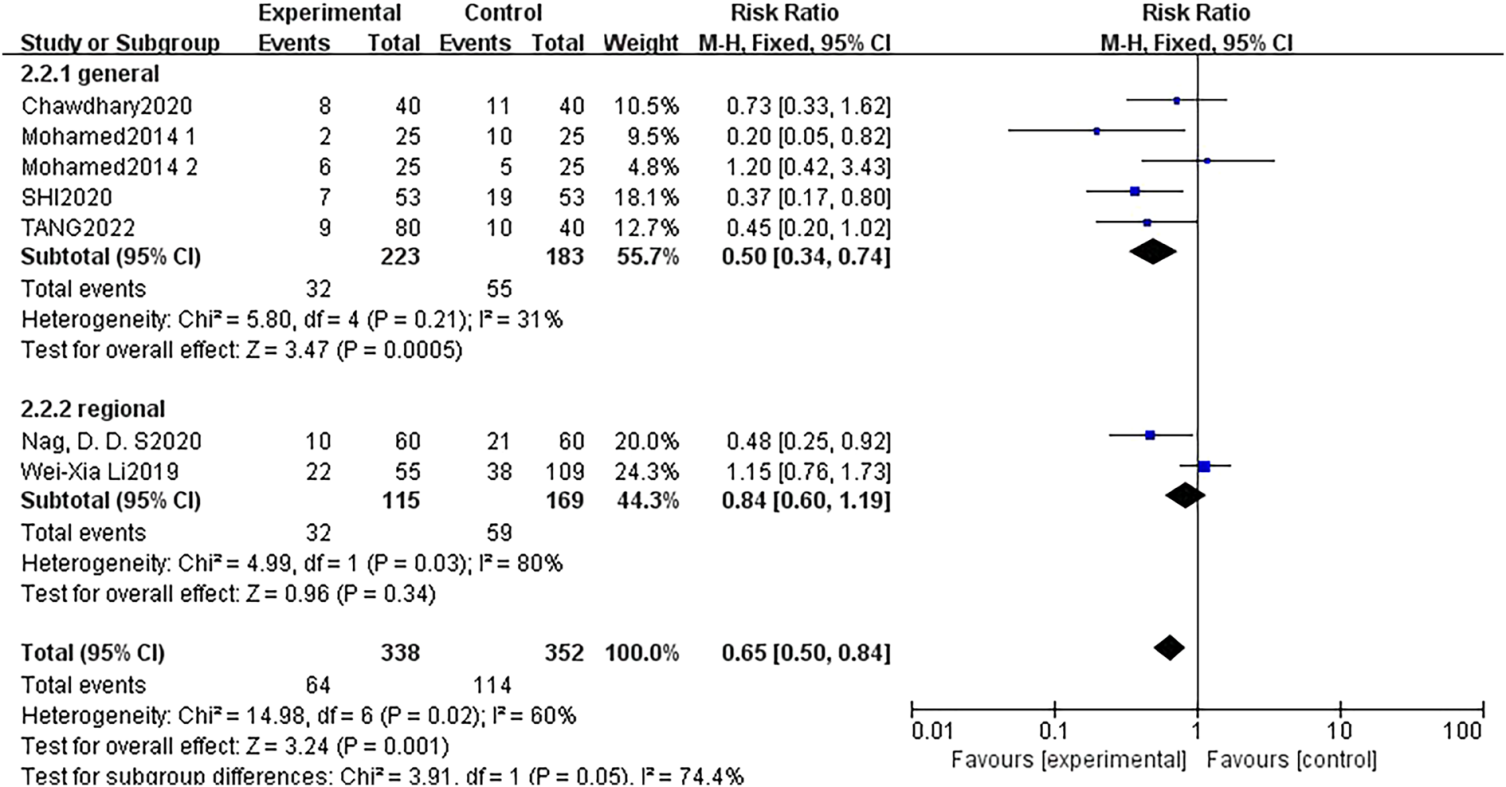
Forest plot of POCD at 7 days after surgery by subgroup based on the type of anaesthesia

For POCD, subgroups were also assigned according to the type of anaesthesia. Five studies were conducted with patients exclusively receiving general anaesthesia, while two studies were conducted under regional anaesthesia plus sedation. These studies assessed the preventive effect of dexmedetomidine on the incidence of POCD. Compared with regional anaesthesia plus sedation, continuous intravenous injection of DEX during general anaesthesia (5 trials, RR: 0.50; 95% CI 0.34 to 0.74, *p* = 0.0005, *I*^2^ = 31%) may be more beneficial in reducing the risk of POCD (2 trials, RR: 0.84; 95% CI 0.60 to 1.19, *p* = 0.03, *I*^2^ = 80%) (Fig. 9). The type of anaesthesia was considered to be a source of obvious heterogeneity (*I*^2^ = 74.4%).

### Level of certainty for outcomes (GRADE)

GRADE profiler was used to assess the level of certainty in the outcomes of the meta-analysis, following the GRADE methodology. The results were categorised and ranked, ranging from low to high certainty, as presented in Table 2.

**Table 2:** Summary of findings table with GRADE assessment quality of evidence

Outcomes	No. of participants (studies)	Relative effect (95% CI)	Risk of bias	Inconsistency	Indirectness	Imprecision	Publication bias	Overall certainty of the evidence (GRADE)
POD	4304 (20 RCTs)	RR: 0.55; 95% CI 0.47 to 0.64	Not serious	Not serious	Not serious	Serious	Not serious	Moderate
POCD	690 (7 RCTs)	RR: 0.74; 95% CI 0.6 to 0.91	Not serious	Serious	Not serious	Serious	Not serious	Low
Hypertension	694 (4 RCTs)	RR: 1.35; 95% CI 0.81 to 2.24	Not serious	Not serious	Not serious	Serious	Not serious	Moderate
Hypotension	994 (7 RCTs)	RR: 1.42; 95% CI 1.08 to 1.86	Not serious	Not serious	Not serious	Serious	Not serious	Moderate
Bradycardia	994 (7 RCTs)	RR: 1.66; 95% CI1.23 to 2.26	Not serious	Not serious	Not serious	Serious	Not serious	Moderate

### Publication bias and sensitivity analysis

**Fig. 10:**
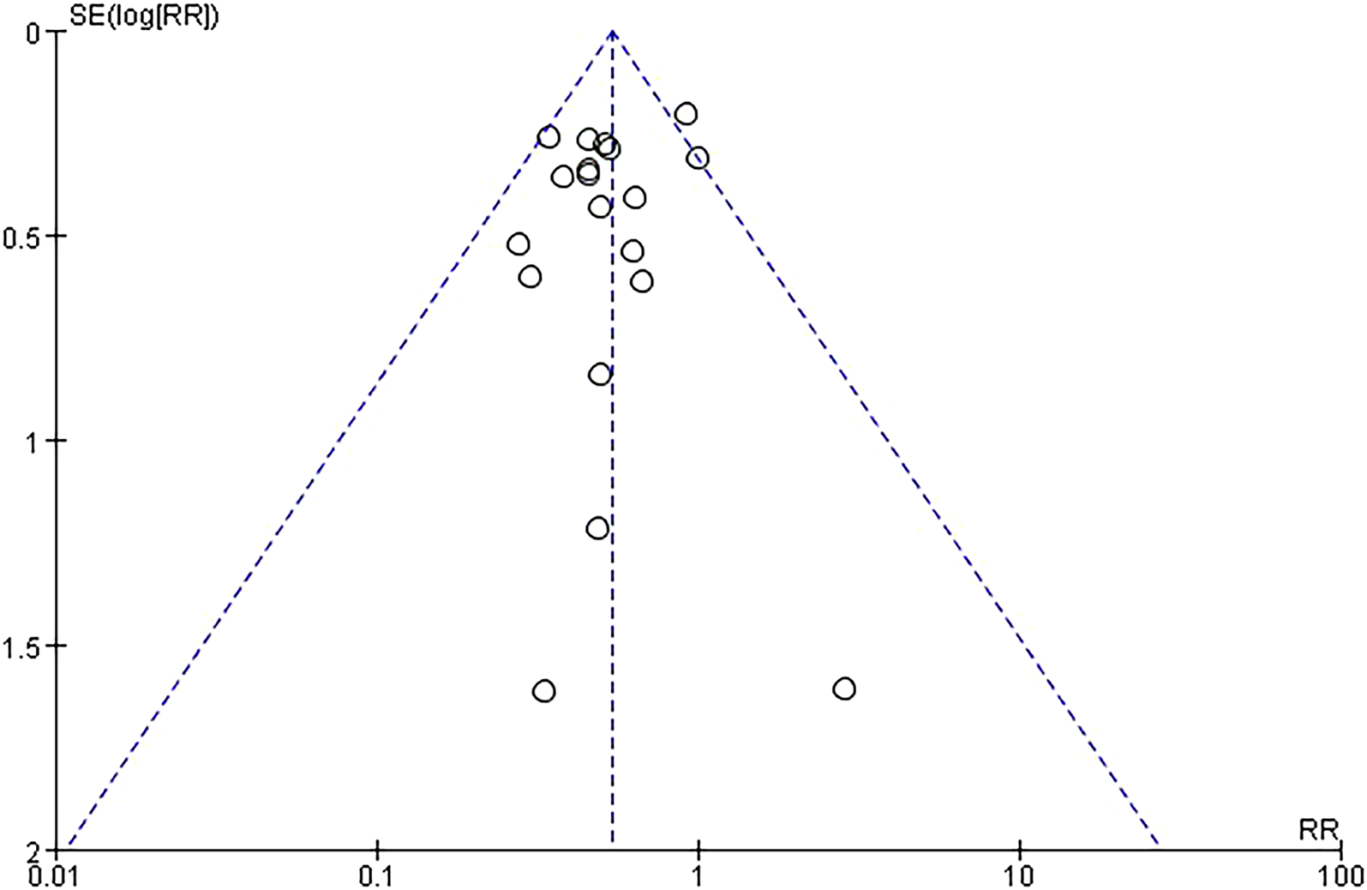
Funnel plot of the incidence of POD

**Fig. 11:**
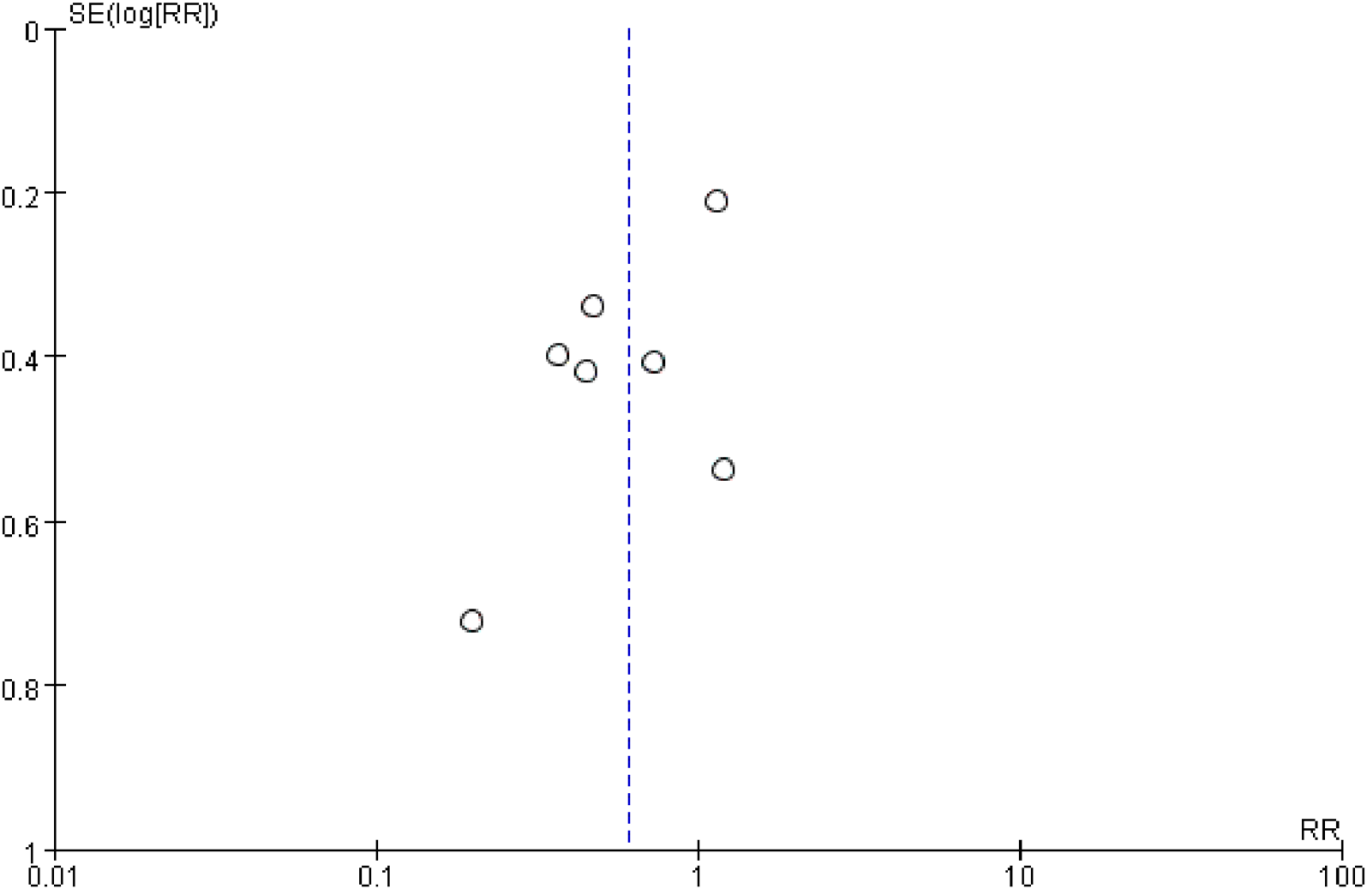
Funnel plot of the incidence of POCD at 7 days after surgery

**Fig. 12:**
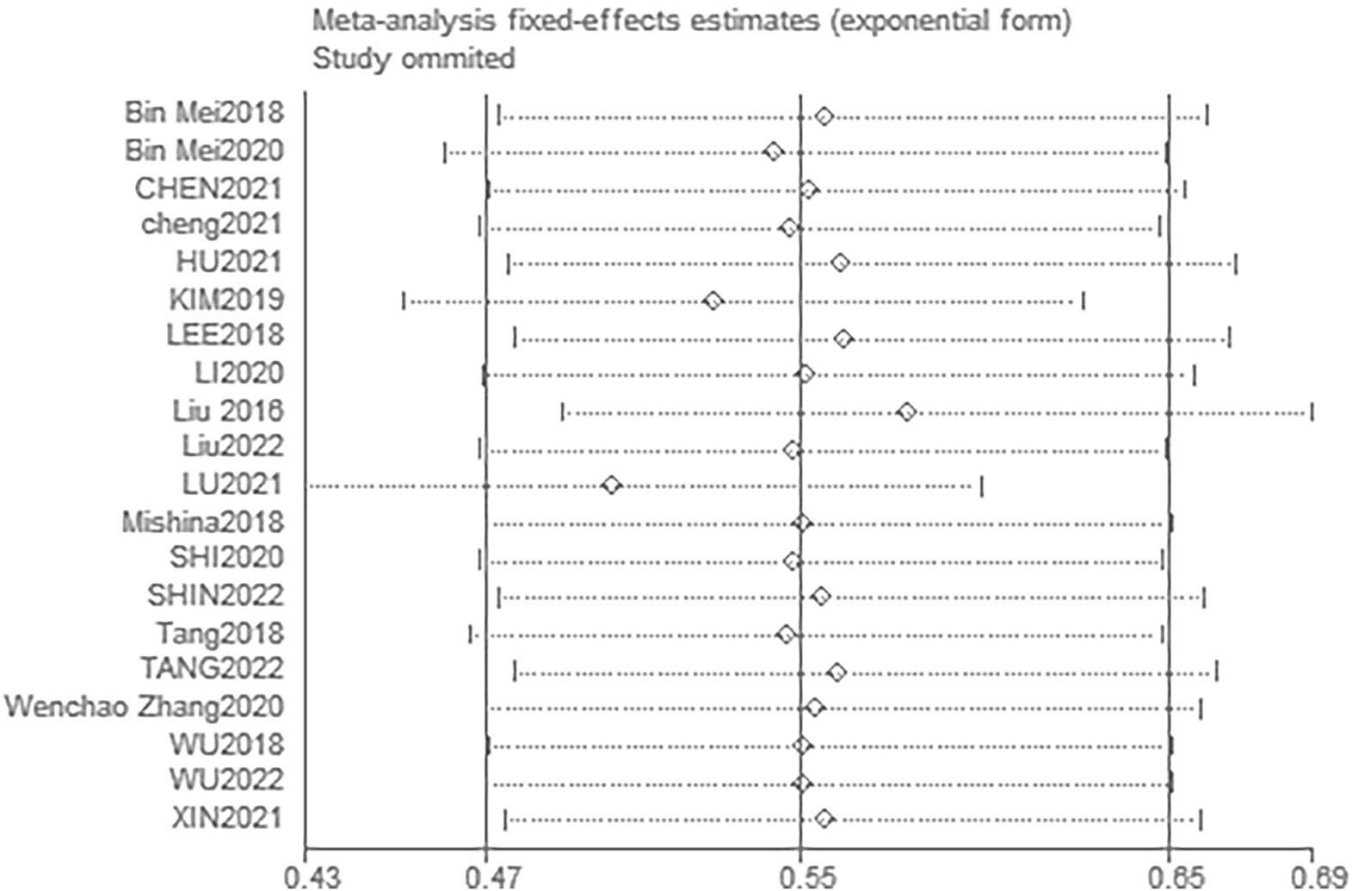
Sensitivity analysis for POD

**Fig. 13:**
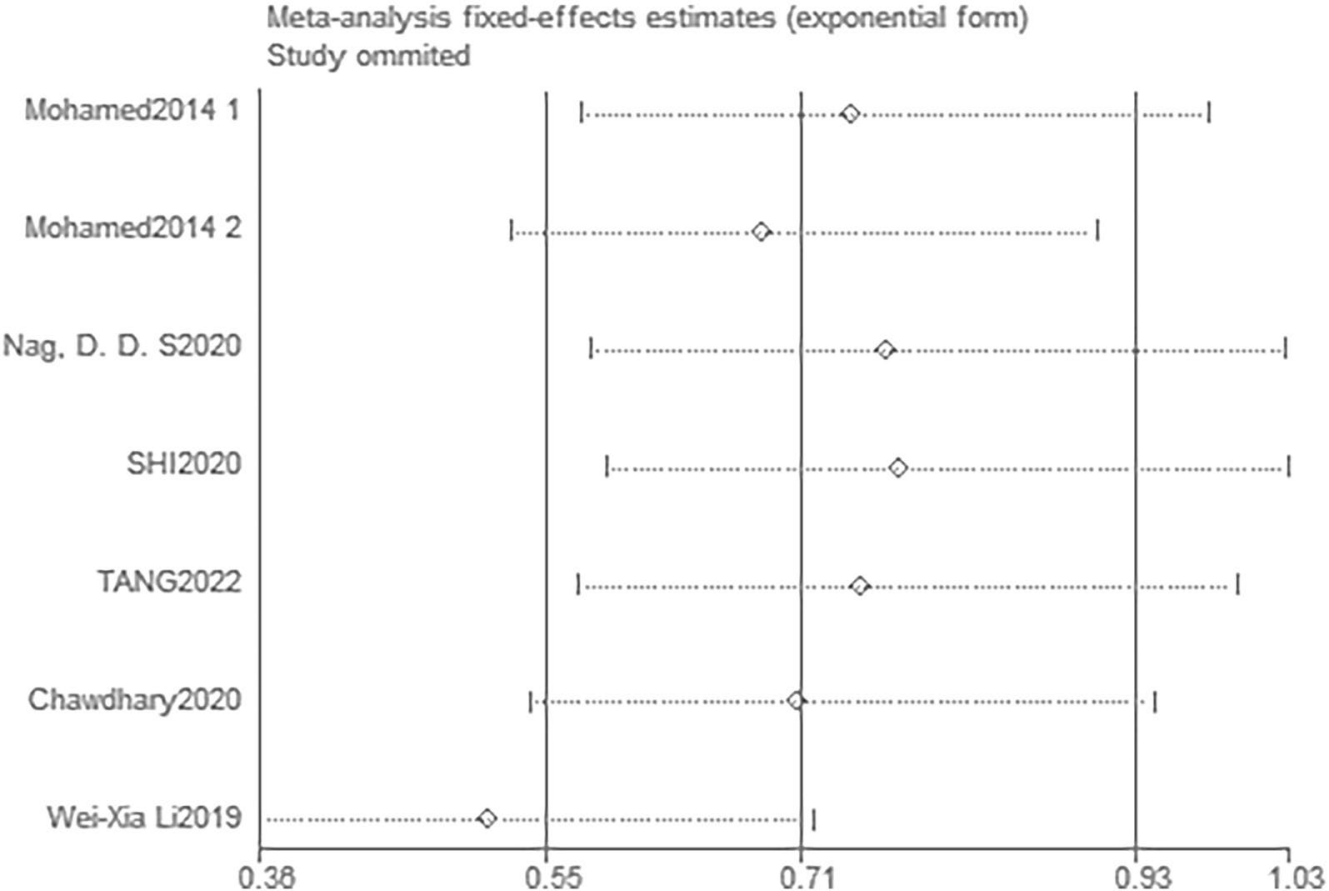
Sensitivity analysis for POCD

The funnel plots (Fig. 10 and 11) and Egger’s tests (with a P value of 0.448 for POD and 0.098 for POCD) suggest that there was no significant publication bias in the studies that investigated the effects of DEX on both POD and POCD. Further, sensitivity analysis was conducted to assess the impact of each individual study on the overall meta-analysis. By excluding one study at a time from the included research, findings were made that the overall outcome regarding POD remained robust (Fig. 12). In the studies on POCD, the exclusion of a particular study [37] significantly altered the results (Fig. 13), and thus was regarded as a source of heterogeneity.

## Discussion

By analysing all available RCTs, intraoperative intravenous DEX infusion was found to reduce the incidence of POD and POCD compared to other sedatives or normal saline. At the same time, the subgroup analysis showed that DEX had a consistent preventive effect on POD in patients undergoing non-cardiac or non-neurosurgery, regardless of the type of anaesthesia used. Meanwhile, another subgroup analysis also suggested that continuous intravenous injection of DEX during general anaesthesia appeared to be more effective in reducing the risk of POCD compared to regional anaesthesia. Despite such results, in contrast to the control group receiving normal saline or other sedatives, patients receiving DEX during surgery were more prone to experiencing hypotension and bradycardia, whereas the occurrence of hypertension during surgery did not differ significantly between the two groups.

DEX demonstrates effectiveness in preventing POD and POCD across various surgical and anaesthesia types. Continuous intravenous DEX administration during general anaesthesia appears to be more effective in reducing the risk of POCD compared to regional anaesthesia. However, clinicians should exercise caution and closely monitor patients for potential side effects when using DEX, without excessive concern for hypertension.

POD and POCD have become major concerns in clinical practice because of their potential to cause prolonged hospital stays, higher medical expenses and lower quality of life for patients. Risk factors for these complications include advanced age, pain, anaemia, and hypoxemia [39]^.^ The use of certain anaesthetic drugs has also been implicated as a risk factor for POD [40–42]. Proposed mechanisms for these complications include the disruption of normal neurotransmitter function, alterations in tau protein, inflammatory responses, disruptions in calcium homeostasis, and impairment of mitochondrial function. Moreover, POD has been recognised as a significant risk factor for adverse outcomes in hip fracture patients. Patients with either POCD or POD have been observed to experience a twofold increase in the risk of mortality [43] Thus, it is crucial to recognise patients who are susceptible to POD and POCD and take preventive measures to mitigate their incidence.

Fortunately, it has been suggested that 40% of POD cases can be prevented [44]. One method of prevention involves administering DEX during the perioperative period. DEX is a highly selective alpha-2 adrenergic receptor agonist known for its neuroprotective effects. DEX does not interact with gamma-aminobutyric acid receptors, lacks anticholinergic activity, and has been demonstrated to promote natural sleep patterns, which could contribute to its potential anti-delirium effects [45]. Several interventions, including multimodal analgesia techniques, cognitive stimulation programs, and early mobilisation, have also been proposed for the prevention of POD and POCD [44].

Two meta-analyses published in 2019 [46] and 2022 [47] were conducted to investigate the impact of DEX on the postoperative neurocognitive function of elderly patients who underwent non-cardiac surgery. Concerning elderly patients who underwent non-cardiac surgery, both studies concluded that administering DEX during the perioperative period was more effective than other sedatives in preserving cognitive function after the surgical procedure. Simultaneously, Zeng H et al. found that DEX also leads to an increased incidence of hypotension and bradycardia. The results of our study are generally consistent with these research findings. However, these meta-analyses only included the elderly population and did not apply to all patients. Although POD and POCD are more common in older patients, they can still occur in relatively young patients and middle-aged people after major surgery. Further, neither meta-analysis included a subgroup analysis based on anaesthetic modality to assess whether the efficacy of DEX in preventing POD and POCD was influenced by differences in the type of anaesthesia. This is a crucial consideration because various anaesthesia types can exert distinct impacts on neurocognitive function. As such, a new meta-analysis was undertaken to overcome these limitations and offer a more comprehensive analysis of the influence of DEX on POD and POCD.

The heterogeneity observed in the analysis of POCD can be primarily attributed to this particular study. An assumption could be made that this heterogeneity may be linked to factors such as the small sample size, a high rate of loss to follow-up, variations in drug administration protocols within the study, and differences in the assessment method for POCD compared to other studies.

In this new meta-analysis, databases were added for up-to-date literature searches, resulting in a considerably larger number of included RCTs and patients compared to previous studies. Additionally, a subgroup analysis was conducted to evaluate the influence of DEX on POD and POCD under different types of anaesthesia. The focus of the meta-analysis was on evaluating the effect of continuous intravenous infusion of DEX during surgery on the prevention of POD and POCD in patients of all age groups, providing a more comprehensive and reliable analysis of the neuroprotective effects of DEX.

The present meta-analysis supports previous findings that the administration of DEX to patients undergoing non-cardiac and non-neurosurgical procedures has a significant beneficial effect on postoperative cognitive function. In parallel, the analysis demonstrates that a continuous intraoperative infusion of DEX is effective in preventing both POD and POCD. The subgroup analysis revealed that DEX had a consistent preventive effect on POD regardless of anaesthesia type, and continuous intravenous injection of DEX during general anaesthesia was more effective in reducing the risk of POCD than regional anaesthesia. These results suggest that DEX could serve as a valuable resource in perioperative care for this patient population. Further research is warranted to explore its potential benefits in future studies.

However, the present meta-analysis demonstrates that DEX leads to bradycardia, hypertension and hypotension, which can be attributed to its activity as an α2-adrenoreceptor agonist. When DEX is administered, it produces a distinct two-phase haemodynamic response, whereby lower plasma concentrations lead to a reduction in blood pressure, while higher plasma concentrations result in an increase in blood pressure [48]. Notably, the hypotension and bradycardia associated with DEX are primarily observed as intraoperative adverse effects. Moreover, there is a paucity of sufficient studies to establish a clear link between these intraoperative adverse effects and long-term postoperative adverse outcomes in patients. It is possible that there may be a superior medication or a novel drug administration strategy that can effectively prevent both POD and POCD while also improving the haemodynamic stability of patients [49]. Further research and development in this area may lead to advancements in perioperative care.

The present study had several limitations and shortcomings. Firstly, it should be emphasised that there was considerable heterogeneity in both the assessment measures employed to evaluate POCD and the types of drugs administered to the control group among the studies incorporated in the analysis. Secondly, more precise studies are needed to determine if there is an optimal dosing regimen and dosage to protect the patient's cognitive function with less haemodynamic impact on the patient. Thirdly, the accuracy of the results was compromised by the under-representation of certain groups of studies. Therefore, in the future, clinicians may conduct more RCTs, with particular focus on various drug administration strategies and the timing of DEX infusion, in order to confirm the impact of different administration methods on POD and POCD.

## Conclusion

Intravenous DEX infusion during surgery reduces POD and POCD risk in elderly non-cardiac, non-neurosurgery patients. Continuous DEX infusion in general anaesthesia is more effective for POCD prevention than regional anaesthesia. DEX is equally effective in POD reduction regardless of anaesthesia type. However, intraoperative hypotension and bradycardia may occur. There might exist an advanced medication or innovative drug delivery approach capable of preventing both POD and POCD while enhancing patients' haemodynamic stability.

## Data Availability

All data generated or analysed during this study are included in this published article.
